# Effects of intranasal instillation of nanoparticulate matter in the olfactory bulb

**DOI:** 10.1038/s41598-021-96593-0

**Published:** 2021-08-20

**Authors:** So Young Kim, Kyung Woon Kim, So Min Lee, Sohyeon Park, Byeong-Gon Kim, Eun-kyung Choi, Bu Soon Son, Moo Kyun Park

**Affiliations:** 1 Department of Otorhinolaryngology, College of Medicine, CHA University, Seongnam, South Korea; 2 Department of Otorhinolaryngology, Seoul National University College of Medicine, Seoul, South Korea; 3 Electron Microscope Lab, Seoul National University Hospital, Seoul, Republic of Korea; 4 Department of Medical Biotechnology, SoonChunHyang University, Asan, Chungnam Republic of Korea; 5 Sensory Organ Research Institute, Medical Research Center, Seoul National University, Seoul, South Korea

**Keywords:** Biochemistry, Molecular biology, Environmental sciences

## Abstract

Nanoparticulate matter activates the aryl hydrocarbon receptor (AhR) pathway in the respiratory system in a process involving the AhR nuclear translocator (ARNT) and cytochrome P450 family 1, member A1 (CYP1A1). We examined changes in AhR-related pathways following intranasal instillation of nanoparticulate matter in the olfactory bulb and cerebral cortex. Twice a day for 5 days per week for 1 week or 2 weeks, 8-week-old Sprague–Dawley rats were intranasally instilled with 10 µL nanoparticulate matter (nano group; n = 36). An equal volume of saline was intranasally instilled in control rats (n = 36). One week after intranasal instillation, olfactory function and Y-maze tests were performed. The expression levels of AhR in the olfactory bulb and temporal cortex were analyzed using western blotting and immunofluorescence assays. The expression levels of *AhR, CYP1A1*, inducible nitric oxide synthase (*iNOS*), and five genes encoding cation transporters (*ARNT*, *ATP7B*, *ATPB1*, *OCT1*, and *OCT2*) in the olfactory bulb were analyzed using quantitative reverse transcription. The olfactory discrimination capability was reduced in the nano group compared with the control group. Proportional changes in the Y-maze test were not significantly different between the nano and control groups. AhR mRNA and protein expression in the olfactory bulb increased 1.71-fold (*P* < 0.001) and 1.60-fold (*P* = 0.008), respectively. However, no significant changes were observed in the temporal cortex. In the olfactory bulb, the expression of *ARNT*, *ATP7B*, *ATPB1*, and *OCT2* was downregulated. *CYP1A1* and *iNOS* expression in the olfactory bulb was upregulated compared with that in the temporal cortex. The intranasal instillation of nanoparticulate matter decreased the olfactory discrimination ability, which was accompanied by upregulation of AhR expression and downregulation of cation transporters in the olfactory bulb.

## Introduction

Hazardous effects of particulate matter have been reported in cases of olfactory dysfunction^1–3^. Children exposed to particulate matter exhibit decreased olfactory sensitivity, and metal particles, including lead particles, likely induce the poor olfactory function associated with particulate-matter exposure^1^. Besides effects on olfaction, the impacts of particulate matter exposure on the central nervous system (CNS) have been described^4,5^. After 4–8 weeks of inhalation of particulate matter, oxidative stress and inflammation in various CNS regions, including the prefrontal cortex, temporal cortex, striatum, and cerebellum, were induced^4^. In addition to inflammatory responses, particulate matter inhalation can cause olfactory dysfunction by negatively impacting neural plasticity^2,5,6^. Long-term inhalation of particulate matter resulted in changes in neurotransmitters and perineuronal nets and reduced spatial ability and olfactory sensitivity^5^. Furthermore, gestational exposure to diesel-exhaust particles induced olfactory dysfunction and disturbed neuromodulatory homeostasis in the olfactory bulb in a rabbit model^2^. Therefore, olfactory dysfunction following particulate matter exposure may be associated with both oxidative stress and neuronal changes, which can impact other CNS regions.

Airborne particulate matter may activate oxidative stress and inflammatory responses via upregulation of the aryl hydrocarbon receptor (AhR), which in turn activates AhR-dependent pathways and induces respiratory diseases, cardiovascular diseases, and atherosclerosis^7–11^. As a receptor of both endogenous and exogenous chemicals such as aromatic hydrocarbons, AhR is a transcriptional regulatory factor belonging to the basic-helix-loop-helix/PeriARNT-Sim family^12^. When ligands including xenobiotics of dioxins or polychlorinated biphenyls bind to AhR, the receptor is translocated into the nucleus by dimerizing with the AhR nuclear translocator (ARNT)^12^. Then, the AhR/ARNT heterodimer binds to xenobiotic response elements and activates the transcription of target genes such as cytochrome P450 family 1, member A1 *(CYP1A1*)^13^. However, little is known about changes in AhR-related pathways in the olfactory bulb and brain regions.

We hypothesized that intranasal instillation of nanoparticulate matter might induce olfactory dysfunction and activate AhR-related pathways, which would then impact the CNS via the olfactory bulb. To test this hypothesis, an olfactory sensitivity test was conducted, and changes in gene expression in AhR-related pathways in the olfactory bulb were evaluated following intranasal instillation of nanoparticulate matter. Olfactory dysfunction related to exposure to nanoparticulate matter may be accompanied by CNS injuries, such as oxidative stress. To investigate associated changes in the CNS, spatial working memory function was assessed using a Y-maze test, and changes in the expression of oxidative stress response genes in the cerebral cortex were examined after nanoparticulate matter exposure. We also examined changes in the temporal cortex, which was revealed to be a cerebral cortical region susceptible to particulate matter inhalation in our previous studies^4,5^.

## Materials and methods

### Animal experiments

**Figure 1:**
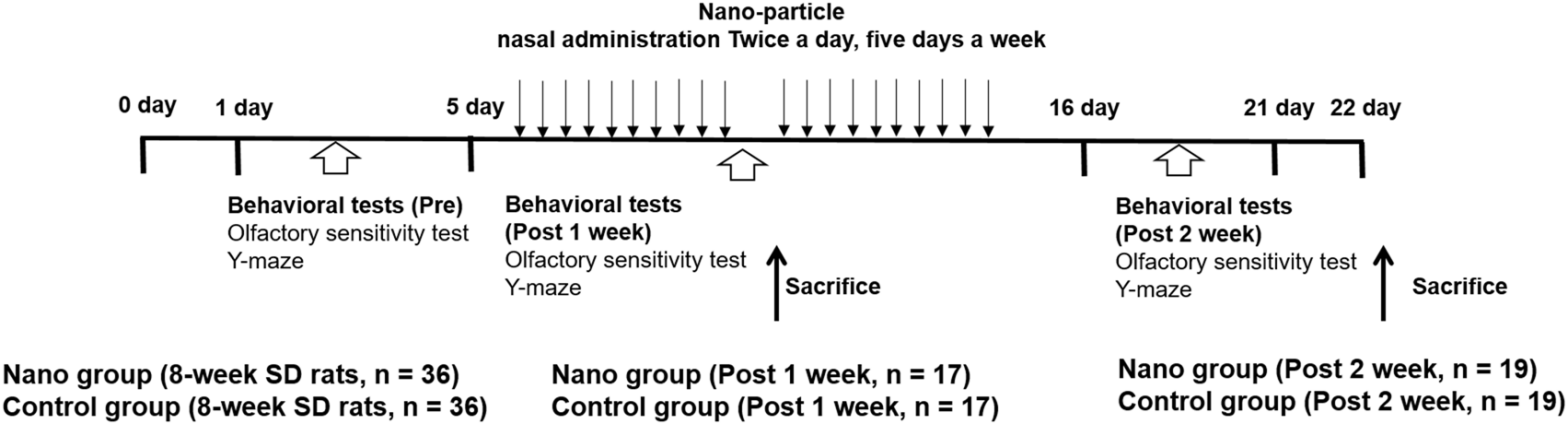
The experimental design of the study. Postnatal 8-week-old female Sprague–Dawley rats were administered nanoparticulate matter (diameter, 0.1–0.056 µm). Two behavioral tests, the olfactory sensitivity test and Y-maze test, were conducted before and after nasal instillation of nanoparticulate matter.

The study was approved by the Institutional Animal Care and Use Committee of CHA University Medical School (Pocheon, Korea; IACUC190046). Study protocol is carried out according to relevant guidelines. Study protocol is carried out according to ARRIVE guidelines. Postnatal 8-week-old Sprague–Dawley rats were divided into nano and control groups (n = 36 per group) (Fig. 1). For each rat in the nano group, 10 µL 53.6 µg/mL nanoparticulate matter (diameter, 0.1–0.056 µm) was administered via intranasal instillation (Fig. 1). In the control group, 10 µL saline was instilled intranasally. Nasal instillation was performed in each rat twice a day for 5 days per week for 1 week (10 times, n = 17 per group) and 2 weeks (20 times, n = 19 per group). No rats died after intranasal instillation.

### Intranasal instillation of nanoparticulate matter

Nanoparticulate matter was collected in an industrial area (Asan, Korea) five times between September 15, 2017 and November 21, 2017 following a previously described protocol^14^. Airborne nanoparticulate matter was collected using Teflon (Polytetrafluoroethylene) filters (Whatman, Maidstone, UK). After the filters were dried in a desiccator for 48 h, the weight of the nanoparticulate matter was measured using a micro balance (CP2P-F; Satorius, Coettingen, Germany). The filters were then baked for 2 h, immersed in deionized water, and sonicated for 1 h. The heavy metal composition of the nanoparticulate matter was subsequently analyzed (Table 1).

**Table 1:** Compositions of heavy metals in particulate matters (0.1–0.056 µm).

Heavy metals	Cd	As	Pb	Cr	Cu	Mn	Ni	Be
Average concentrations (pg/2.72 µg)	2.22	0.10	2.51	16.60	7.14	2.68	6.39	0.00
Standard deviation	4.97	0.23	3.37	35.26	9.38	2.59	10.23	0.00

### Olfactory sensitivity tests

Olfactory sensitivity tests were conducted before and after the instillation of nanoparticulate matter (nano group) or saline (control group) as described previously (n = 36 per group)^5^. First, all rats were placed in an examination cage for 30 min for habituation. Square filter papers measuring 2 × 2 in were impregnated with distilled water (control scent), 10% peanut butter solution (attractive scent), or 10% trichloroacetic acid solution (aversive scent). Rats were exposed to each scent for 3 min. In between sessions, the rats rested in another clean empty cage for 6 min. The total number of sniffs and total duration of sniffing by each rat were recorded.

### Testing spatial working memory using a Y-maze

Spatial working memory was tested before and after instillation of nanoparticulate matter or saline using a Y-maze with a width of 11 cm, length of 50 cm, height of 30 cm, and arms spaced 120° apart, as described previously (n = 36 per group)^5^. Briefly, each rat was placed at the distal end of the start arm and allowed to explore the maze freely for 5 min. Entries into new arms were considered alternations. A rat was considered to enter an arm when it crossed the midpoint of the arm. The number of total arm entries and the percentage of spontaneous alternations were recorded. The alternation percentage (%) was calculated using the formula: (number of successful alternations/[total arm entries − 2]) × 100.

All rats were sacrificed after the tests, and the olfactory bulbs and temporal cortices were harvested. The tissues of 68 rats (n = 17 per group of post 1 week exposure, n = 17 per group of post 2 week exposure) were frozen for further analyses. Brain tissues were harvested for quantitative real-time reverse-transcription polymerase chain reaction (qRT-PCR) and western blotting. The brains of the remaining four rats were fixed via immersion in 4% paraformaldehyde solution for immunofluorescence analysis.

### Transmission electron microscopy

The administration of nanoparticulate matters was examined in the olfactory bulb using transmission electron microscope (TEM). TEM (JEM-1400, Japan) was used as our previous study^15^. The olfactory bulb tissue was prepared and immersion-fixed. The tissue was washed in deuterated H_2_O_2_ and then dehydrated in ethanol solutions. The tissue was treated with propylene oxide and EPON epoxy resin (Embed 812, Nadic methyl anhydride, poly Bed 812, dodecenylsuccinic anhydride, and dimethylaminomethyl phenol, Electron Microscopy Polysciences, USA). The samples were sectioned in 65 nm using an ultramicrotome (RMC MT-XL). The sections were imaged by TEM at 80 kV.

### Determining mRNA expression levels using qRT-PCR

Analysis by qRT-PCR was performed as described previously^4^ and met the requirements regarding the minimum information needed to publish the results of qRT-PCR experiments^16^. Nine rats per group were used for qRT-PCR analysis. Within 4 h after tissue harvest, total RNA from each tissue was extracted using TRIzol reagent (Invitrogen, Carlsbad, CA, USA). Reverse transcription was performed using Maxime RT PreMix (Oligo (dT)15 Primer) (iNtRON Biotechnology, Seongnam, Korea) according to the manufacturer’s instructions. The purity and quantity of the purified RNA were checked by measuring the 260/280-nm absorbance ratio using the Micro UV–Vis spectrophotometer (Lifereal Biotechnology Co. Ltd., Hangzhou, China). Only samples with a 260/280 ratio > 1.8 and a 260/230 ratio > 1.5 were used for qRT-PCR. *AhR, CYP1A1,* inducible nitric oxide synthase (*iNOS*), ARNT, ATP synthase subunit A 1 (*ATPB1*), ATPase copper transporting beta (*ATP7B*), organic cation transporter (*OCT*) 1, and *OCT2* were reverse transcribed and amplified by PCR using forward and reverse primers (Table 2) and TOPrea qPCR 2 × PreMix (SYBR Green with low ROX; Enzynomics, Daejeon, Korea) on the ViiA7 Real-time PCR system (Applied Biosystems, Foster City, CA, USA). The following protocol was used for qRT-PCR: initial activation of HotStarTaq^®^ DNA polymerase at 95 °C for 15 min, followed by 50 cycles of 95 °C for 10 s, 60 °C for 15 s, and 72 °C for 15 s. The amplification efficiency (E) of each amplicon was determined using tenfold serial dilutions of positive control cDNA and calculated from the slopes of the log input amounts (from 20 ng to 2 pg cDNA), which were plotted according to the crossing-point values using the formula E = 10^–1/slope^. All primer efficiencies were confirmed to be high (> 90%) and comparable. The calculated mRNA levels were normalized to those of glyceraldehyde 3-phosphate dehydrogenase according to the formula 2^–Ct^ and expressed as a percentage of the reference gene.

**Table 2:** Oligonucleotide primer sequences for quantitative reverse transcriptase polymerase chain reaction.

Gene	Primer sequence (forward)	Primer sequence (reverse)	Annealing temperature (°C)	Product size (bp)	RefSeq number
*CYP1A1*	5'-AACCTGGGTTCCCAAAGGTC-3'	5'-TCAGTGACAGGTGTGGGTTC-3'	60	212	NM_012540.3
*AhR*	5'-CTCCCTCCACAGTTGGCTTTGTTTG-3'	5'-GATTCTGCGCAGTGAAGCATGTCAG-3'	60	233	NM_001308255.1
*iNOS*	5'-AGGCCACCTCGGATATCTCT-3'	5'-TCTCTGGGTCCTCTGGTCAA-3'	60	85	NM_012611.3
*ARNT*	5'-GGCCAGCTATAGTCATTCCCA-3'	5'-CTCGGATCTCTGTCCTGCAC-3'	60	114	NM_012780.3
*ATP7B*	5'-CTGCAAAGAGGAACTCGGGA -3'	5'-AGTCTGGGGACCTGTACCTT-3'	60	181	NM_012511.2
*ATPB1*	5'-CCAAACGTCCTACCTGTCCA-3'	5'-CATAGAATCCGCCCATCCCA-3'	60	94	NM_013113.2
*OCT1*	5'-CATCTGTGTCCGGTGTGCTA-3'	5'-CTGGTACAAAATGGCCGTCG-3'	60	167	NM_012697.1
*OCT2*	5'-ATCGCAGAATGGTGGGGATT-3'	5'-GCCATCTTGGAGATTCCGGT-3'	60	171	NM_031584.2

### Assaying the AhR protein expression level

Western blotting was performed as described previously^4^. Eight rats per group were used for western blotting. PRO-PREP protein extraction solution (Intron Biotechnology, Korea) was used to extract protein. The protein concentration was checked using a Bio-Rad Protein Assay Kit. Approximately 20 μg protein were separated using 12% sodium dodecyl sulfate–polyacrylamide gel electrophoresis and transferred to polyvinylidene difluoride membranes (Merck Millipore, Burlington, MA, USA). Membranes were soaked in blocking buffer (5% non-fat dry milk in Tris-buffered saline containing Tween-20) for 1 h at room temperature and then incubated with anti-AhR primary antibody (mouse monoclonal; Santa Cruz Biotechnology, Santa Cruz, CA, USA) and β-actin (D6A8, rabbit mAb; Cell Signaling Technology, Danvers, MA, USA). Immunoreactive proteins were detected using a horseradish peroxidase (HRP)-coupled secondary antibody (anti-rabbit IgG, HRP-linked antibody; Cell Signaling Technology) and visualized using an enhanced chemiluminescence kit (Bio-Rad Laboratories, Hercules, CA, USA). Protein bands were quantified via densitometry using ImageJ gel analysis software (National Institutes of Health, Bethesda, MD, USA). Protein expression levels were normalized to those of β-actin.

### Immunofluorescence staining of AhR protein

Immunofluorescence staining was performed as described previously^4^. Two rats per group were used for the analysis. The olfactory bulb and temporal cortex from each rat were dehydrated and embedded in paraffin using optimal cutting temperature solution. Then, 10-µm sections of embedded tissue were cut using a rotary microtome and mounted on glass slides. Each slide was dipped in xylene for 10 min for paraffin removal and sequentially washed in a 100%, 75%, and 50% ethanol series (5 min per wash). The free-floating sections were then washed in phosphate-buffered saline (PBS) three times (5 min per wash). After washing, the sections were placed in 10% goat or donkey blocking serum (Vector Labs, Burlingame, CA, USA) for 1 h at room temperature. The free-floating sections were then incubated overnight at 4 °C on a shaking table with an anti-AhR primary antibody (1:200, mouse monoclonal; Santa Cruz Biotechnology). The following day, the sections were washed in PBS three times (10 min per wash) and incubated with a secondary antibody (1:200, Alexa Fluor 594 donkey anti-mouse, #A21203; Invitrogen) for 2 h at room temperature. The olfactory bulb and primary auditory cortex (AP, − 5 mm; L, 7 mm; V, 3.1–4.9 mm) were analyzed and photographed using a fluorescence microscope (ECLIPSE Ni-U; Nikon Corporation, Tokyo, Japan).

### Statistical analysis

The data are presented as means ± standard error. Means were compared between groups using Student’s t-test after testing for normality using the Shapiro–Wilk test in SPSS software (ver. 21.0; IBM Corp., Armonk, NY, USA). Statistical significance was defined as *P* < 0.05.

## Results

**Figure 2:**
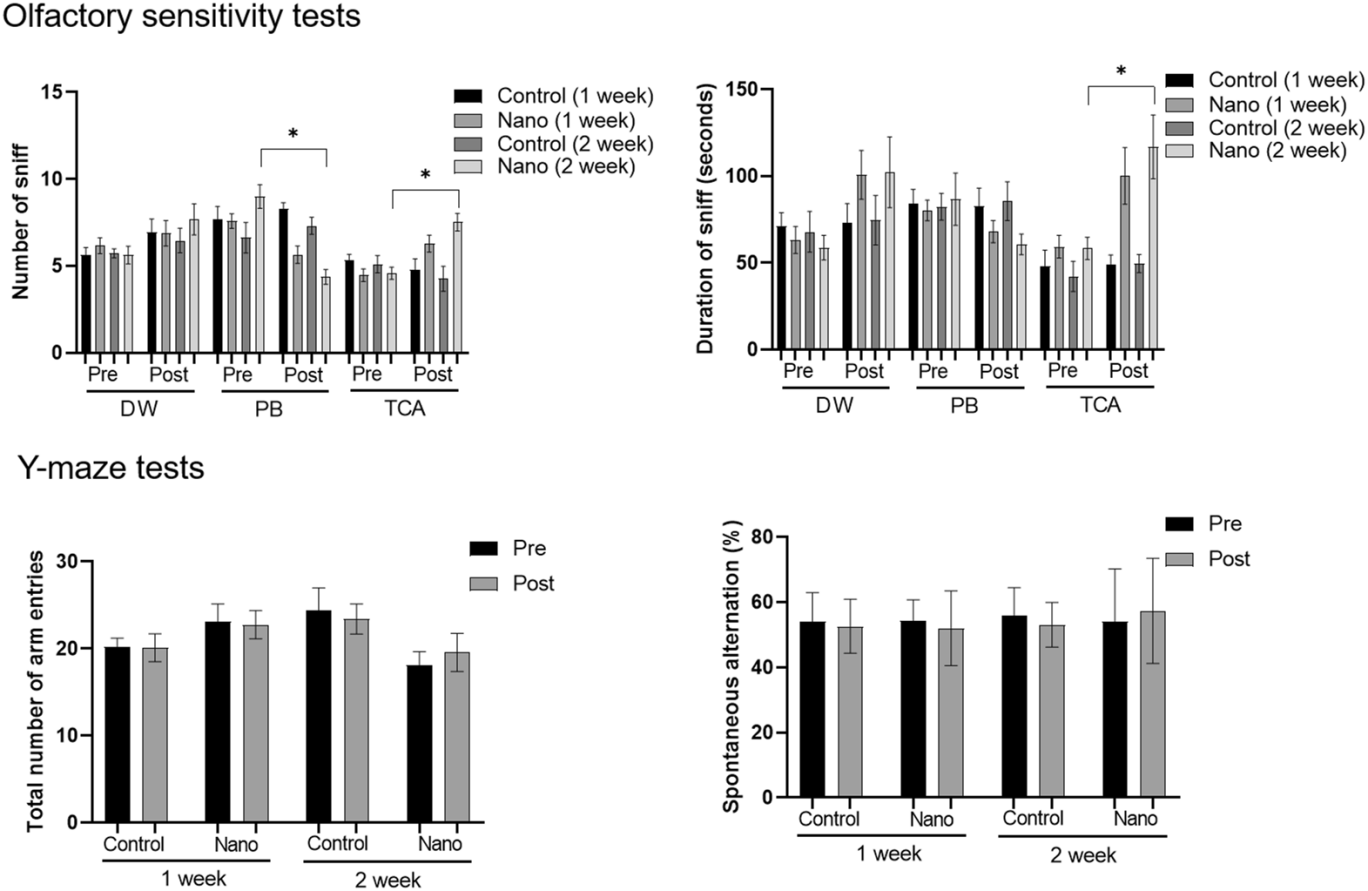
Changes in the frequency and duration of sniffing in the olfactory sensitivity test as well as total number of arm entries and percentage of spontaneous alternations in the Y-maze test after exposure to nanoparticulate matter. In the nano group (n = 17 per group for 1-week exposure and n = 19 per group for 2-week exposure), the number of sniffs decreased for the attractive scent [peanut butter (PB)] and increased for the aversive scent [trichloroacetic acid (TCA)] after exposure to nanoparticulate matter. *, *P* < 0.05, paired t-test, pre- vs. post-exposure.

In the control group, the frequency and duration of sniffing did not differ between pre- and post-1 week and 2 week of intranasal instillation in rats exposed to the control (distilled water), attractive (peanut butter), or aversive (trichloroacetic acid) scents (Fig. 2). In the nano group, both the frequency and duration of sniffing the attractive scent (peanut butter) decreased from pre-exposure to post 2-week exposure to nanoparticulate matter (number of sniffs: 9.00 ± 0.70 vs. 4.37 ± 0.43; *P* < 0.001; duration of sniffing: 86.81 ± 15.06 vs. 60.69 ± 6.01 s; *P* = 0.116). By contrast, the rats in the nano group sniffed the aversive scent (TCA) more frequently post 2-week exposure to nanoparticulate matter (pre-exposure, 4.58 ± 0.35 sniffs; post-exposure, 7.53 ± 0.50 sniffs; *P* < 0.001), whereas the duration of sniffing the aversive scent (TCA) increased post-exposure (pre-exposure, 58.38 ± 6.43 s; post-exposure, 116.89 ± 18.36 s, *P* = 0.005).

**Figure 3:**
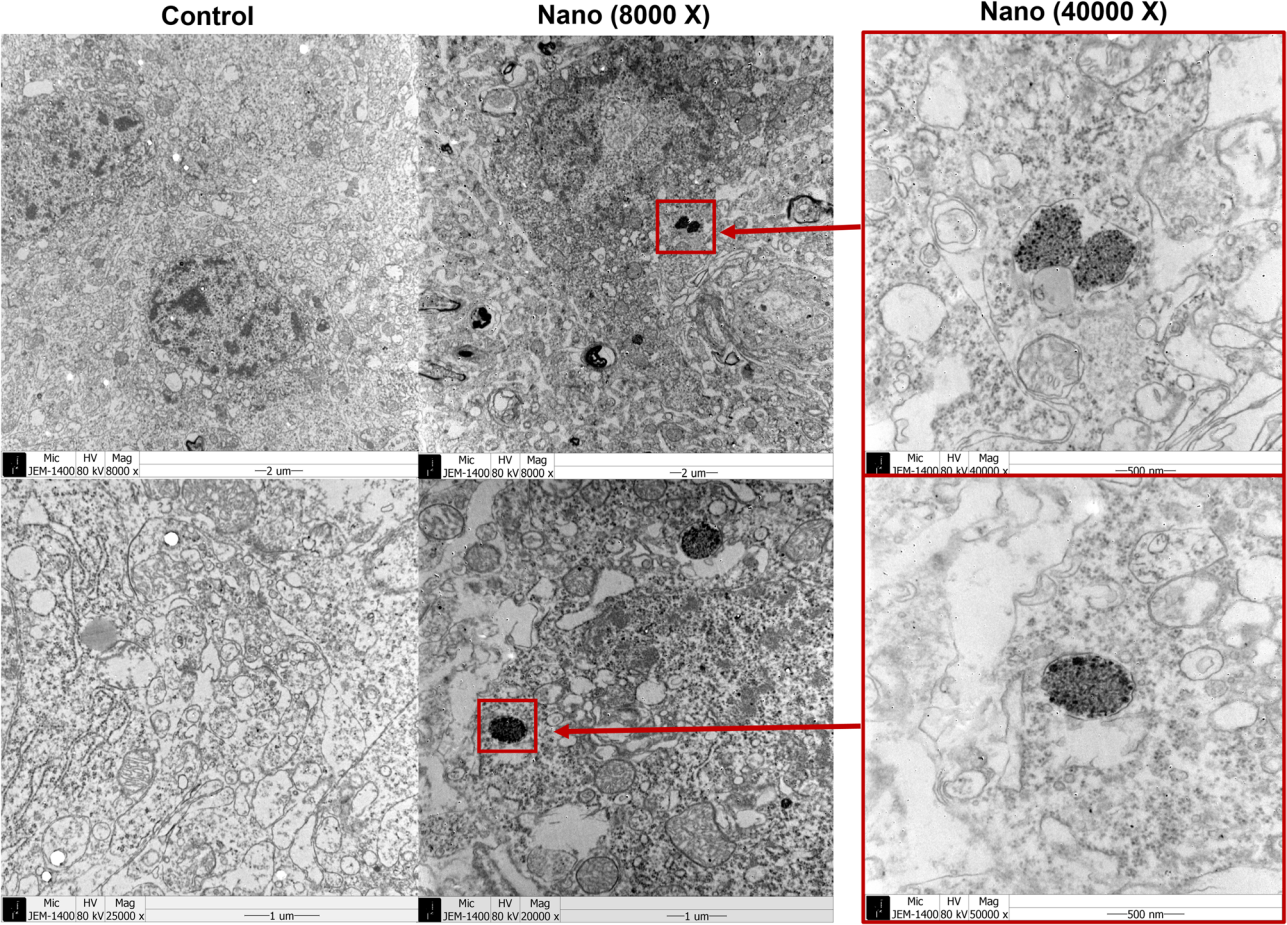
Transmission electron microscopic findings of olfactory bulbs of control and nano groups. The aggregates of electron-dense particles (red arrows) were identified in the olfactory bulb of nano group, which was not detected in that of control group.

In the Y-maze test, the total number of arm entries and the spontaneous alternation percentage did not differ significantly between the control and nano groups both pre- and post-intranasal instillation (all *P* > 0.05) (Fig. 2). The aggregates of electron-dense particles were detected in olfactory bulb (Fig. 3). They were scattered in intracellular regions of around nucleus and vesicles. In the olfactory bulb of control group, there was no electron-dense aggregated in TEM examinations.

**Figure 4:**
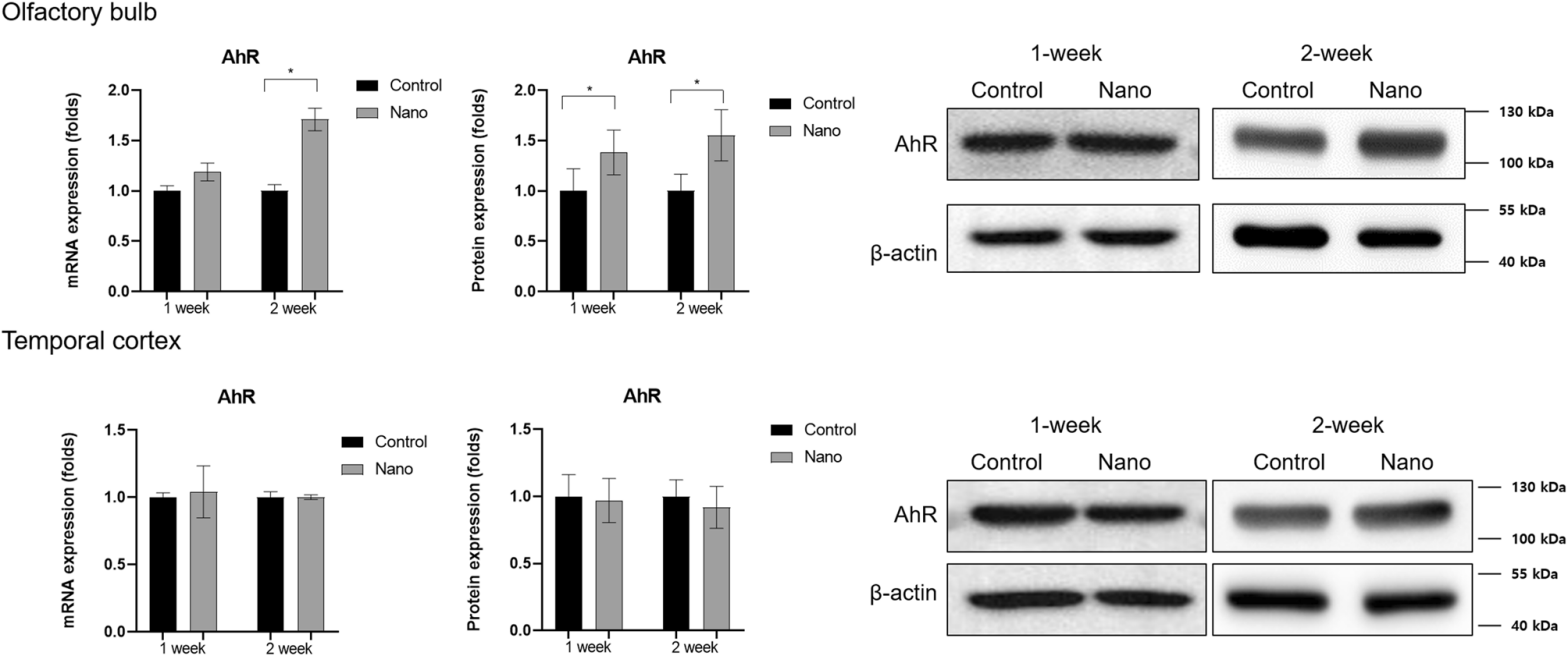
Comparisons of protein and mRNA expression levels of the aryl hydrocarbon receptor (AhR) in the olfactory bulb and temporal cortex between the control and nano groups (n = 8 per group for western blotting and n = 9 per group for qRT-PCR). Both protein and mRNA expression levels of AhR increased in the olfactory bulb in the nano group post-exposure. *, *P* < 0.05, unpaired t-test, control vs. nano group.

**Figure 5:**
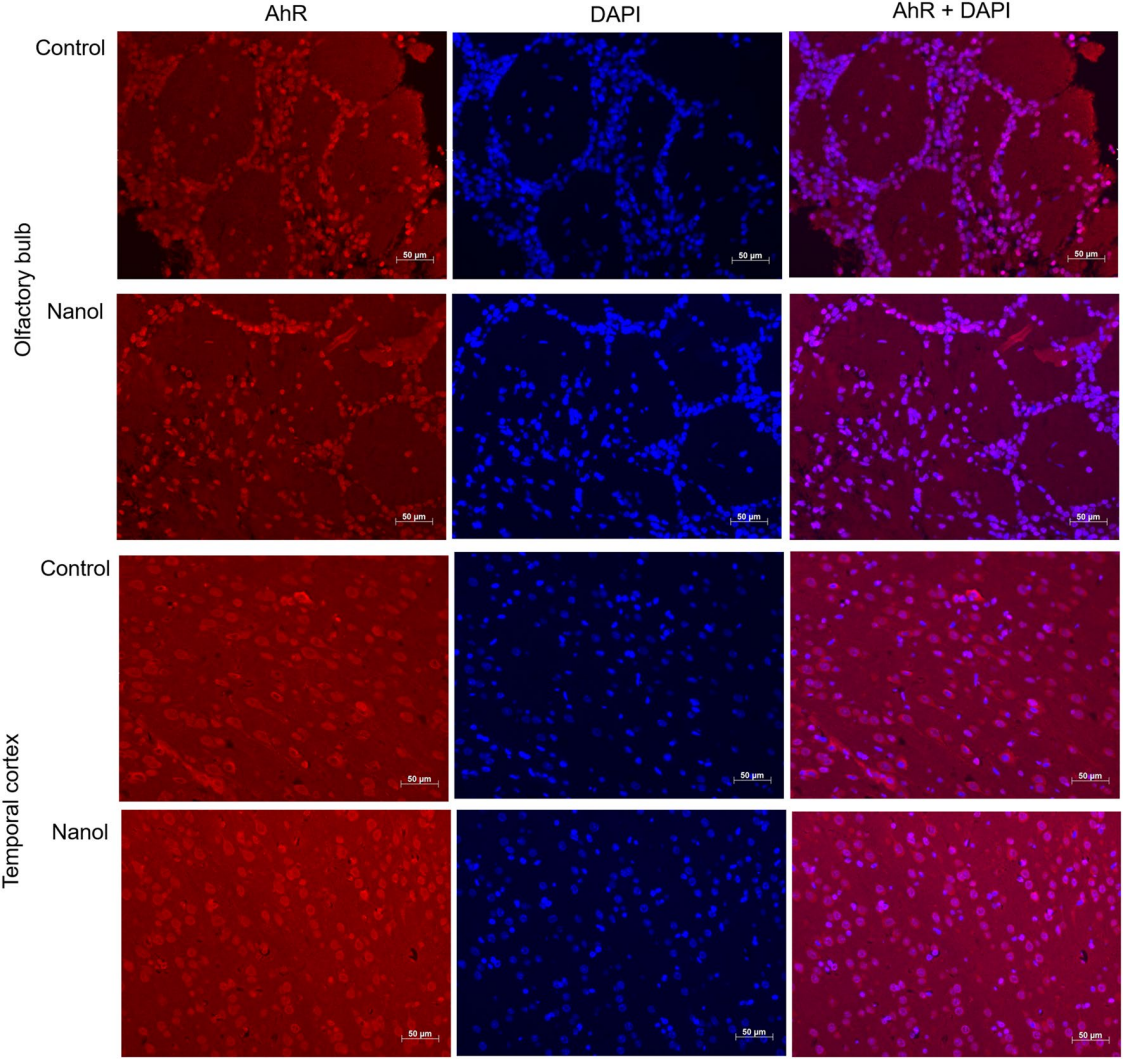
Immunofluorescence analysis of AhR expression. The AhR protein level (red) in the olfactory bulb was increased in the nano group compared with the control group (blue, DAPI).

In the olfactory bulb, *AhR* mRNA was expressed at higher levels in the nano group than in the control group after 2-week exposures (fold change, 1.71 ± 0.11; *P* < 0.001) (Fig. 4, Table S1). AhR protein expression levels were also higher after 1-week and 2-week nano exposures (fold change, 1.69 ± 0.23; *P* = 0.047 for 1-week groups and 1.60 ± 0.09; *P* = 0.008 for 2-week groups) (Fig. 4, Fig. S1). In the temporal cortex, *AhR* mRNA and protein expression levels did not differ between the nano and control groups. Immunofluorescence staining showed tendencies of more AhR-positive cells in the olfactory bulb in the nano group compared with the control group, although statistical significance could not be delineated due to the small number of specimens (Fig. 5).

**Figure 6:**
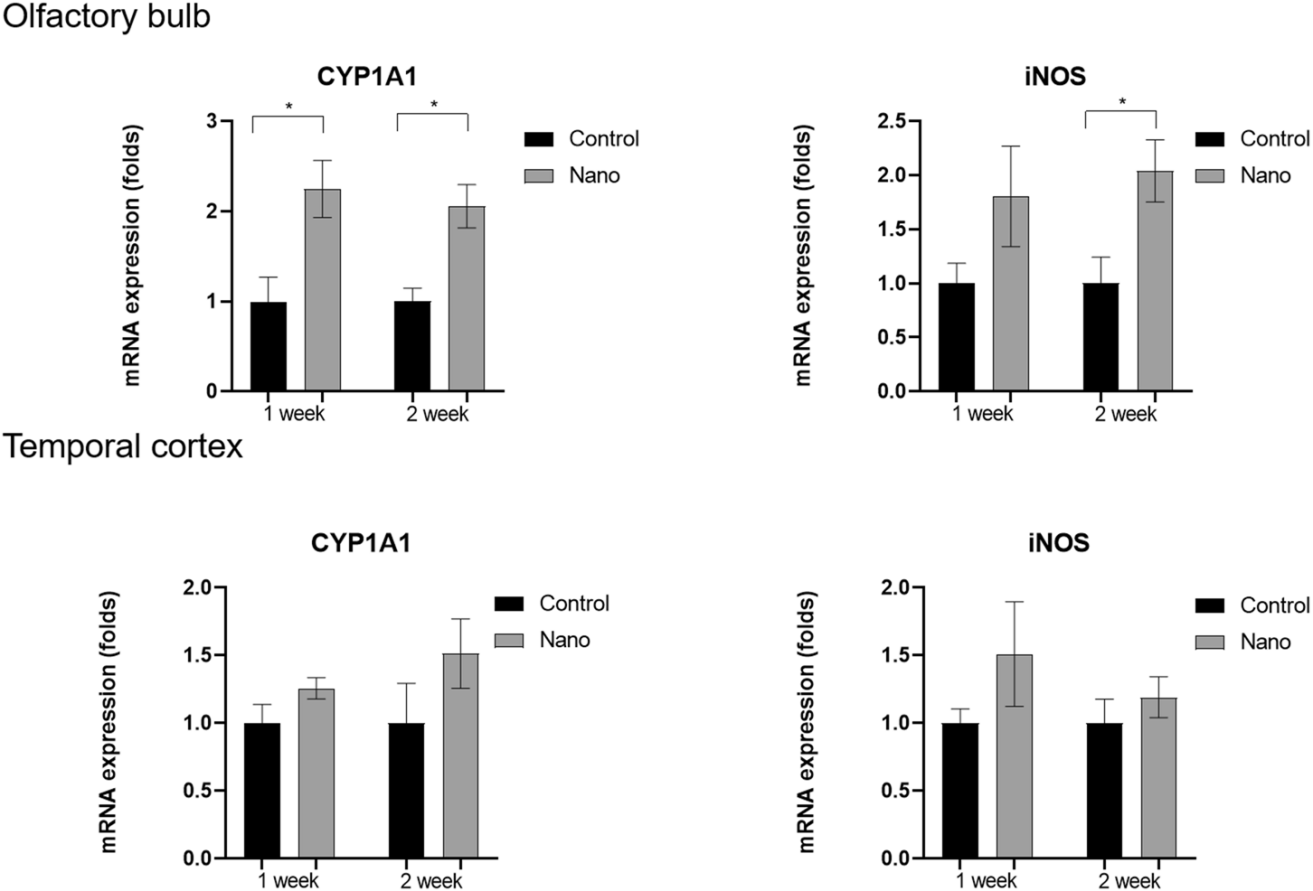
Comparisons of the mRNA expression levels of *CYP1A1* and *iNOS* in the olfactory bulb and temporal cortex between the control and nano groups (n = 9 per group). Both *CYP1A1* and *iNOS* expression levels were increased in the olfactory bulb in the nano group. In the temporal cortex, the mRNA expression level of *CYP1A1* was increased in the nano group compared with the control group. *, *P* < 0.05, unpaired t-test, control vs. nano group.

*CYP1A1* expression levels in the olfactory bulb were higher in the 1-week and 2-week nano groups than in their control groups (fold change, 2.25 ± 0.32; *P* = 0.009 for 1-week nano group and 2.06 ± 0.24; *P* = 0.002 for 2-week nano group). *iNOS* mRNA expression levels in the olfactory bulb were higher in the 2-week nano group, but not in the 1-week nano group, than in the control group (fold change, 2.04 ± 0.29; *P* = 0.014) (Fig. 6). *CYP1A1* and *iNOS* mRNA expression levels in the temporal cortex were not upregulated in the 2-week nano group (*CYP1A1* fold change, 1.51 ± 0.26; *P* = 0.206; *iNOS* fold change, 1.19 ± 0.15; *P* = 0.423).

**Figure 7:**
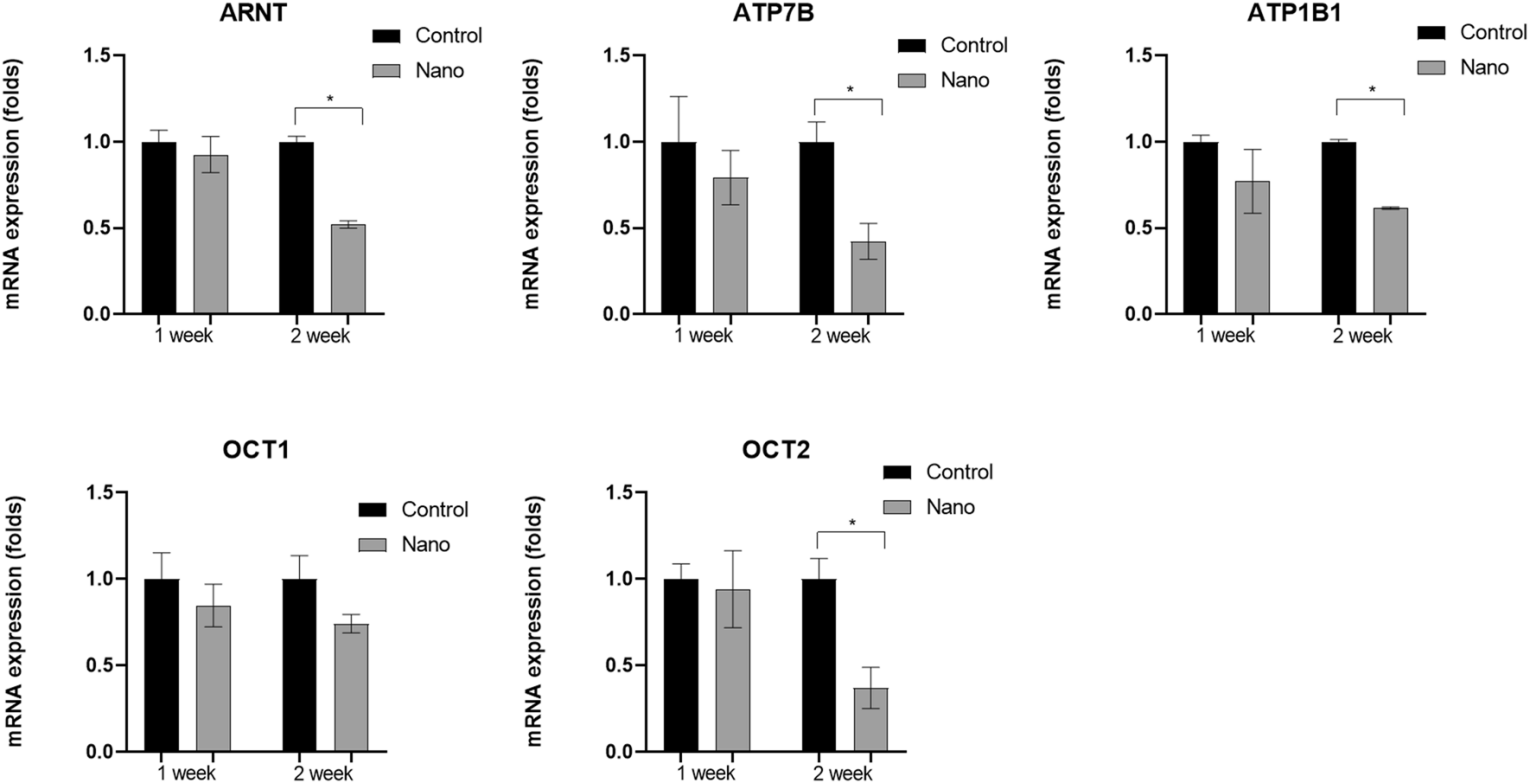
Comparisons of the mRNA expression levels of five genes encoding cation transporters (*ARNT, ATP7B, ATP1B1, OCT1,* and *OCT2*) in the olfactory bulb between the control and nano groups. *ARNT, ATP7B, ATP1B1,* and *OCT2* expression levels were increased in the olfactory bulb in the nano group compared with the control group (n = 9 per group). *, *P* < 0.05, unpaired t-test, control vs. nano group.

To investigate the factors associated with olfactory dysfunction and AhR upregulation in the olfactory bulb, the mRNA expression levels of *ARNT* and four other genes encoding cation transporters (*ATP7B, ATPB1, OCT1*, and *OCT2*) were analyzed (Fig. 7). Lower mRNA expression levels of *ARNT* (fold change, 0.52 ± 0.02; *P* < 0.001), *ATP7B* (fold change, 0.42 ± 0.10; *P* = 0.002), and *ATPB1* (fold change, 0.62 ± 0.01; *P* < 0.001), and *OCT2* (fold change, 0.37 ± 0.12; *P* = 0.002) were found in the 2-week nano group compared with the control group.

## Discussion

The intranasal instillation of nanoparticulate matter upregulated *AhR* expression in the olfactory bulb compared with control group. Similarly, the mRNA expression level of *CYP1A1*, an AhR target gene, was elevated in the olfactory bulb in the nano group compared with control group. In the olfactory bulb, genes related to oxidative stress (*CYP1A1* and *iNOS*) were upregulated. However, genes related to oxidative stress and *AhR* expressions were not upregulated in the temporal cortex compared with control group. Thus, AhR-related oxidative responses may be stronger in the olfactory bulb than in the temporal cortex, and nanoparticulate matter may induce oxidative stress in the CNS largely via the olfactory bulb. AhR upregulation and oxidative stress responses were accompanied by a decrease in olfactory sensitivity in the nano group. The 2-week nanoparticulate matter exposures decreased the number of sniff for favorable scent (peanut butter), while they increased the number of scent and duration of sniff for aversive scent (TCA). The mRNA expression levels of genes encoding cation transporters decreased in the nano group compared to those of control groups, implying that olfactory transduction had decreased in the rats with the instillation of nanoparticulate matter. These changes of *iNOS, ARNT,* and cation transporters (*ATP7B, ATPB1*, and *OCT2*) were existed after 2-week nanoparticulate matter exposures, but not in the 1-week nanoparticulate matter exposures. Olfactory discrimination was also decreased only after 2-week nanoparticulate matter exposures. These implied the dose-dependent impacts of nanoparticulate matters on olfactory bulb and temporal cortex.

AhR is activated following exposure to airborne particulate matter^10,17,18^. In addition to upregulation of *AhR* expression, *CYP1A1* and *iNOS* mRNA levels in rats also increased after exposure to nanoparticulate matter in this study. In line with the present results, oxidative stress and inflammation have been shown to be induced by AhR upregulation^17,19^. In an in vitro study, exposure to e-cigarette aerosols and particulate matter upregulated the expression of AhR, CYP1A1, and inflammatory cytokines^17^. The activation of AhR then influences the olfactory system via oxidative stress responses. Microarray analyses showed that both oxidative stress responses and AhR-related pathways were induced in salmon exposed to organophosphate pesticides and experiencing olfactory dysfunction^20^.

Although we did not observe definitive cognitive dysfunction in rats exposed to nanoparticulate matter, *CYP1A1* and *iNOS* mRNA expression levels were elevated in the temporal cortex after exposure. Because the rats were exposed to nanoparticulate matter only for a short duration of time, long-term exposure may be required to observe obvious cognitive behavioral changes. Accordingly, impaired cognitive functions have been reported following chronic exposure to particulate matter^5,21^. Exposure to fine particulate matter for 3–12 months led to cognitive dysfunction accompanied by neuroinflammatory responses in mice^21^. In our previous study, mice exhibited reduced spatial activity and olfactory sensitivity after 4 weeks of inhalation of diesel-extracted particles^5^. Therefore, olfactory dysfunction may serve as an initial marker of oxidative stress in the CNS following exposure to nanoparticulate matter.

Conversely, exposure to nanoparticulate matter decreased the expression of cation transporters in the olfactory bulb. Olfactory dysfunction in rats exposed to nanoparticulate matter may be linked to disturbances in olfactory transduction via transporter- and AhR-related neuronal injuries. The reduction in transporter function might have impaired olfactory transduction. According to previous in vitro studies, AhR activation after the administration of air pollutants led to decreased expression of transporters including OCT1^22,23^. Cigarette-smoke condensates and diesel-exhaust particles activated AhR cascades and suppressed the mRNA expression of genes encoding transporters including OCT1 in primary human hepatocytes^22,23^. OCTs are strongly expressed in the olfactory bulb and olfactory nerve as well as in other brain regions including the cerebral cortex^24^. Because signaling transduction cascades play a role in olfactory transduction^25–27^, the downregulation of these transporters may result in olfactory dysfunction. In addition, excessive AhR signaling exerts detrimental effects on neurodevelopment in the olfactory bulb, which can bring about olfactory dysfunction following exposure to nanoparticulate matter^28^. Constitutively active AhR signaling has been shown to interfere with cell migration and neurite growth in the olfactory bulb of mice^28^. The mRNA expression of *ARNT*, a cofactor of AhR activation, decreased in the olfactory bulb of rats after exposure to nanoparticulate matter. Mechanisms distinct from those of AhR may regulate ARNT expression. *ARNT* mRNA expression levels are negatively correlated with CYP1A/1B and AhR expression levels, implying that *AhR* and *ARNT* transcription is regulated via different means, and CYP1A/1B-mediated signaling pathways negatively regulate *ARNT*^29^.

In this study, we used an in vivo model to demonstrate the acute effects of exposure to nanoparticulate matter on the olfactory bulb and cerebral cortex and the accompanying changes in behavior. The 2-week exposure was chosen based on our previous study which demonstrated the oxidative stress changes after 2-week exposure to fine particulate matters^15^. The 8-week old (young adult) rats were chosen to exclude the influence of developmental stages and aging processes. In these young adult rats, the nanoparticulate matter exposure induced inflammatory changes of olfactory bulb and temporal cortex without cognitive decline in behavioral tests. Thus, it can be presumed that the inflammatory changes of olfactory bulb and temporal cortex after nanoparticulate matter exposures are initiated ahead of cognitive changes. Moreover, the effects of nanoparticulate matter exposure were higher in longer exposure groups, which implied the dose-dependent effects of nanoparticulate matters on central nervous system. However, due to some limitations, the present results should be interpreted with caution. Due to the small number of samples for immunofluorescence staining, statistical significance could not be concluded for the Ahr- positive cell densities. To estimate the quantitative changes of Ahr, western blots and qRT-PCRs were conducted, which showed increased levels of Ahr in nano groups. In addition, the genes related with oxidative stress and cation transporters were examined in mRNA levels, but not in protein levels. The changes of protein levels for these genes and additional genes using proteomic approach may warrant to delineate the molecular mechanism on the effects of nanoparticulate matter exposure. Nanoparticulate matter was collected from the field and used in clinical exposure experiments. However, we did not assess the detailed contents of the nanoparticulate matter and thus could not separately evaluate the effects of each component on olfactory function and the CNS. The considerable variation observed in the qRT-PCR results might have been partially due to the heterogeneous content of the nanoparticulate matter used in this study. According to an in vitro study, differences in metal oxide (Fe_2_O_3_, ZnO, CeO_2_, Fe_3_O_6_, Al_2_O_3_, CuO, and TiO_2_) composition in nanoparticulate matter can lead to variation among PCR replicates^30^. Metals are presumed to be the main cause of olfactory dysfunction related to particulate matter exposure^1^. Hence, we provided information on the metal composition of the nanoparticulate matter used in this study. Further studies are needed to evaluate nanoparticulate matter composition in greater detail.

## Conclusion

Nasal instillation of nanoparticulate matter decreased olfactory sensitivity and downregulated the expression of cation transporters in the olfactory bulb. AhR-related pathways, including AhR and *CYP1A1* expression, were upregulated in the olfactory bulb. *CYP1A1* expression also increased in the temporal cortex, but no cognitive dysfunction was observed, implying that nanoparticulate-matter exposure can have latent impacts on the CNS.

## Supplementary Information

Supplementary Information.

## Data Availability

The raw data of experiments used to support the findings of this study are available from the corresponding author upon request.
