# Supplementary material for: Effects of intranasal instillation of nanoparticulate matter in the olfactory bulb
Source: Sci Rep. 2021 Aug 20;11:16997. doi: 10.1038/s41598-021-96593-0 (PMC8379193; doi:10.1038/s41598-021-96593-0)

**Effects of intranasal instillation of nanoparticulate matter in the olfactory bulb**


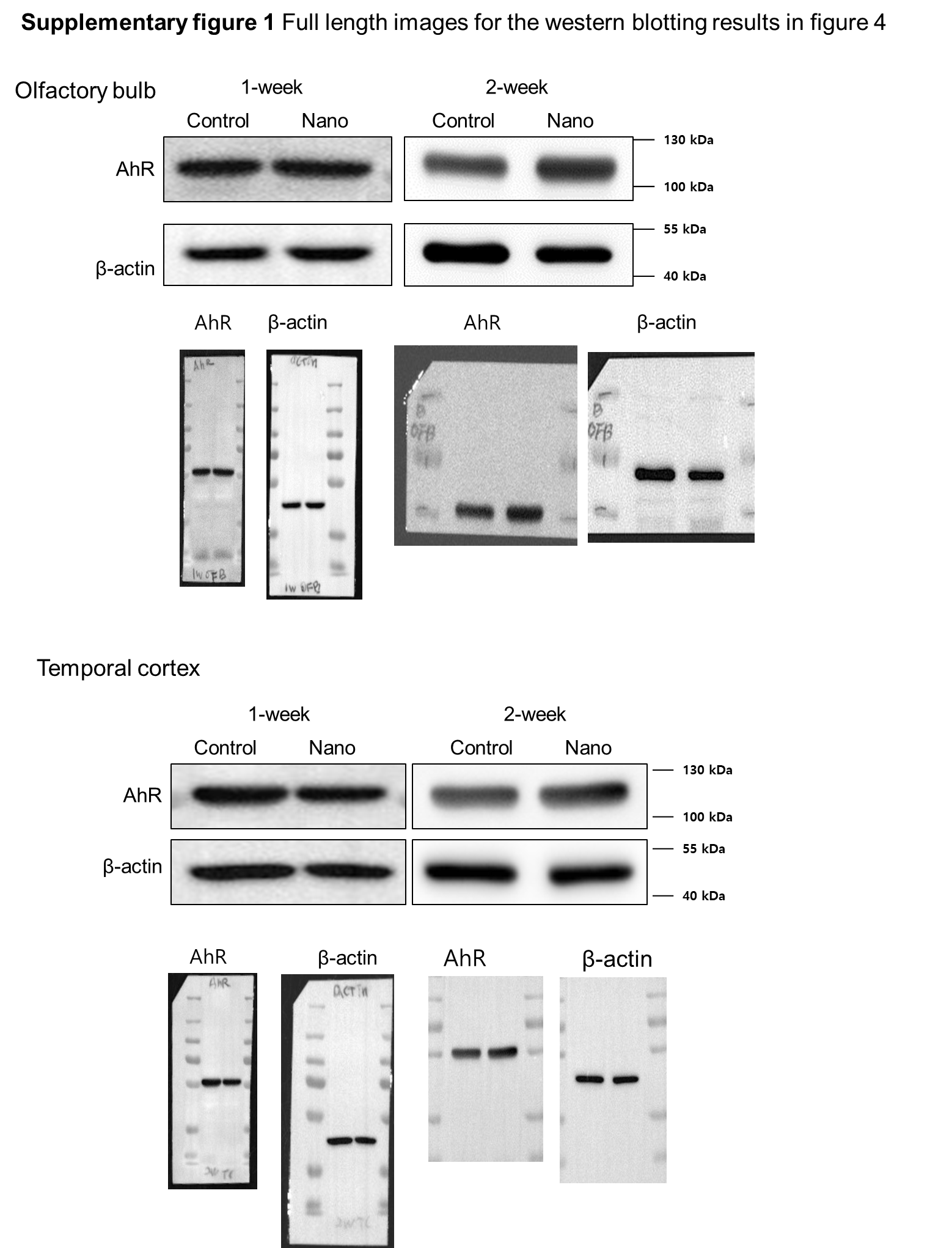


**Supplementary table S1** The gene expression levels in western blotting and qRT-PCRs

|  |  | 1-week exposure | | | | | 2-week exposure | | | | |
| --- | --- | --- | --- | --- | --- | --- | --- | --- | --- | --- | --- |
| Region | Gene | Control | | Nano | |  | Control | | Nano | |  |
|  |  | Mean | SE | Mean | SE | *P*-value | Mean | SE | Mean | SE | *P*-value |
| Olfactory bulb | AhR (protein) | 1.00 | 0.22 | 1.69 | 0.23 | 0.047 | 1.00 | 0.17 | 1.60 | 0.09 | 0.008* |
|  | *AhrR* | 1.00 | 0.05 | 1.19 | 0.09 | 0.083 | 1.00 | 0.06 | 1.71 | 0.11 | <0.001* |
|  | *CYP1A1* | 1.00 | 0.27 | 2.25 | 0.32 | 0.009* | 1.00 | 0.15 | 2.06 | 0.24 | 0.002* |
|  | *iNOS* | 1.00 | 0.19 | 1.81 | 0.46 | 0.127 | 1.00 | 0.24 | 2.04 | 0.29 | 0.014* |
|  | *ARNT* | 1.00 | 0.07 | 0.93 | 0.10 | 0.559 | 1.00 | 0.03 | 0.52 | 0.02 | <0.001* |
|  | *ATP7B* | 1.00 | 0.26 | 0.79 | 0.16 | 0.507 | 1.00 | 0.11 | 0.42 | 0.10 | 0.002* |
|  | *ATPB1* | 1.00 | 0.04 | 0.77 | 0.19 | 0.243 | 1.00 | 0.01 | 0.62 | 0.01 | <0.001* |
|  | *OCT1* | 1.00 | 0.15 | 0.85 | 0.12 | 0.444 | 1.00 | 0.14 | 0.74 | 0.05 | 0.096 |
|  | *OCT2* | 1.00 | 0.09 | 0.94 | 0.22 | 0.811 | 1.00 | 0.12 | 0.37 | 0.12 | 0.002* |
| Temporal cortex | AhR (protein) | 1.07 | 0.16 | 1.00 | 0.16 | 0.756 | 1.00 | 0.12 | 0.92 | 0.11 | 0.628 |
|  | *AhrR* | 1.00 | 0.03 | 1.04 | 0.19 | 0.837 | 1.00 | 0.04 | 1.00 | 0.02 | 0.977 |
|  | *CYP1A1* | 1.00 | 0.14 | 1.26 | 0.08 | 0.128 | 1.00 | 0.29 | 1.51 | 0.26 | 0.206 |
|  | *iNOS* | 1.00 | 0.11 | 1.51 | 0.39 | 0.220 | 1.00 | 0.18 | 1.19 | 0.15 | 0.423 |

SE: standard error, *P < 0.05 (n = 8 per group for western blotting and n = 9 per group for qRT-PCR)

**Supplementary figure S2 Full length gel images for the western blotting**


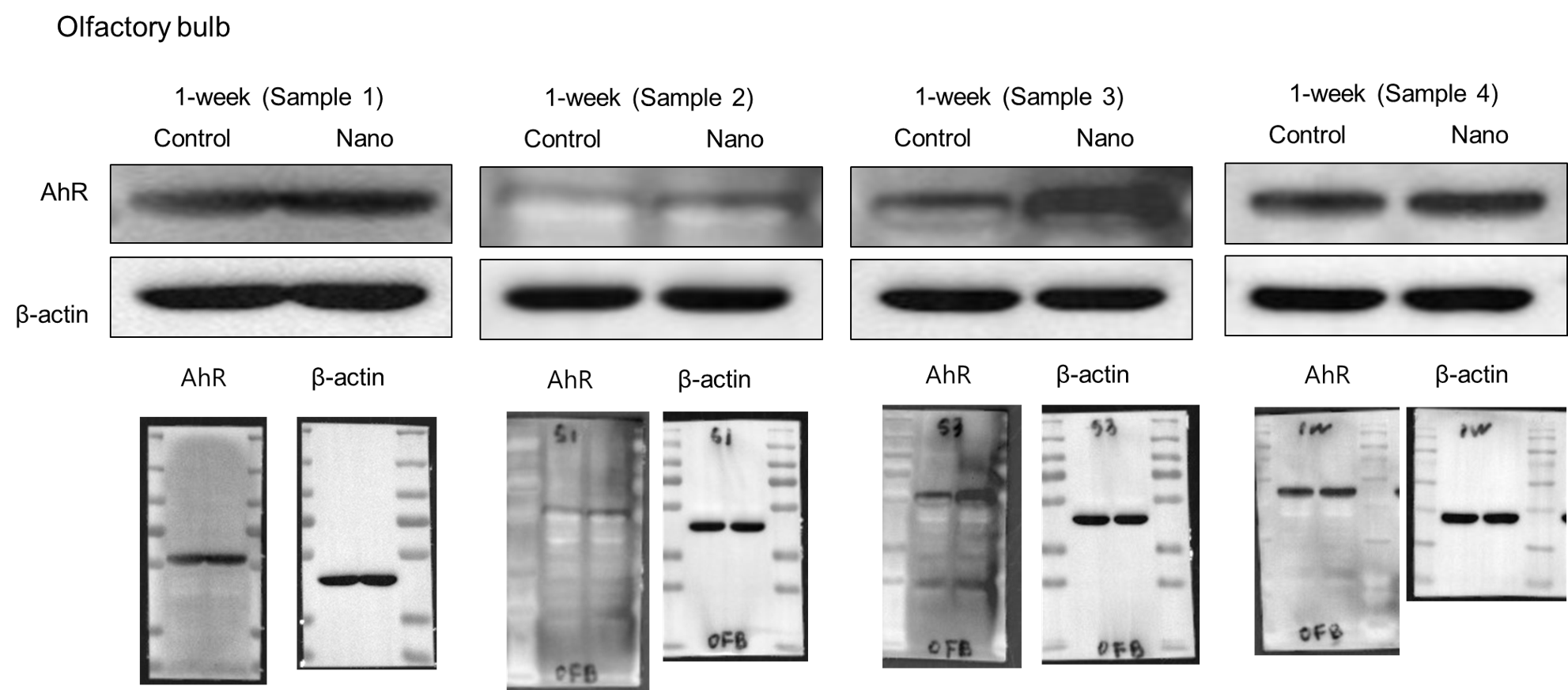

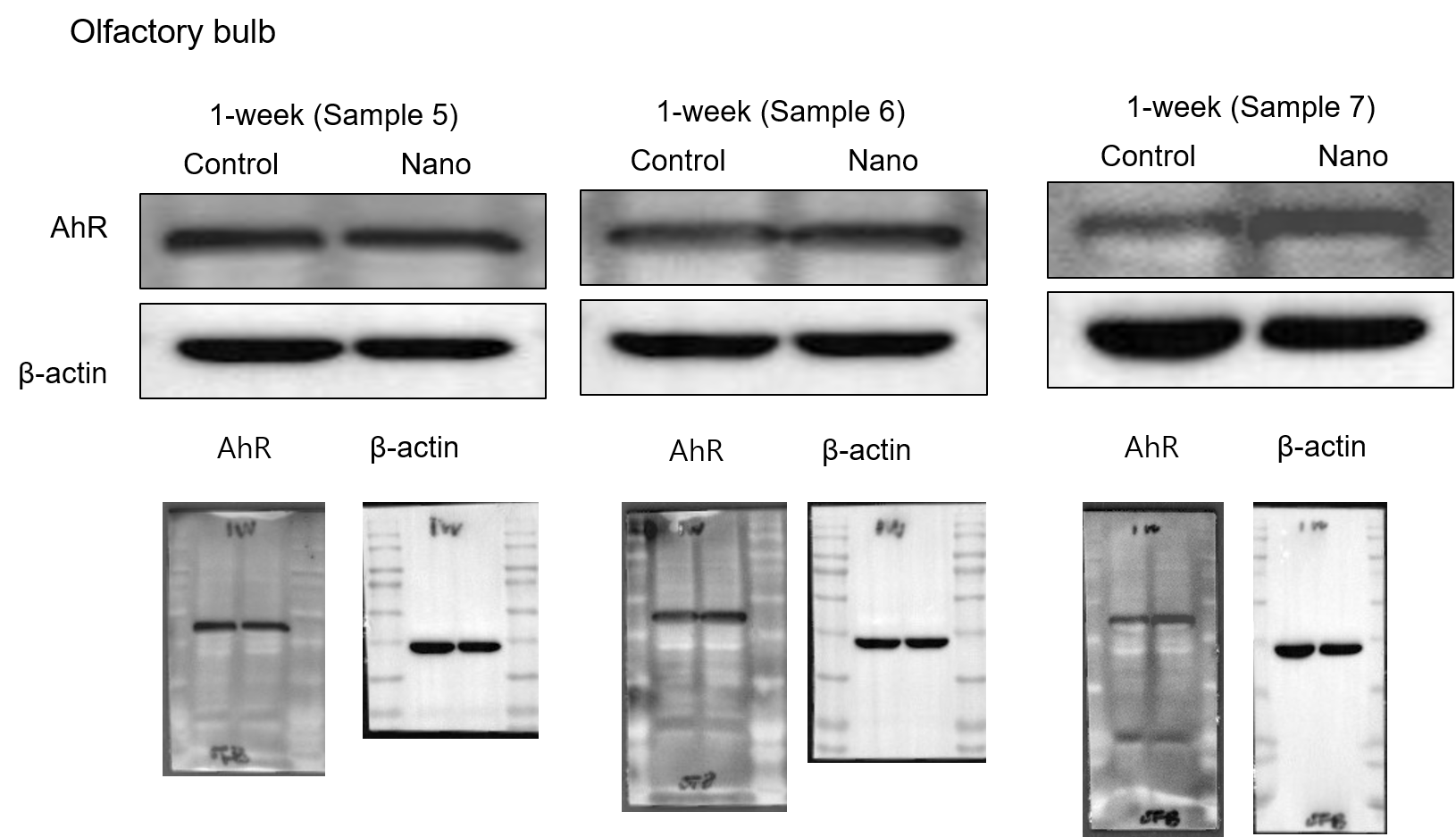

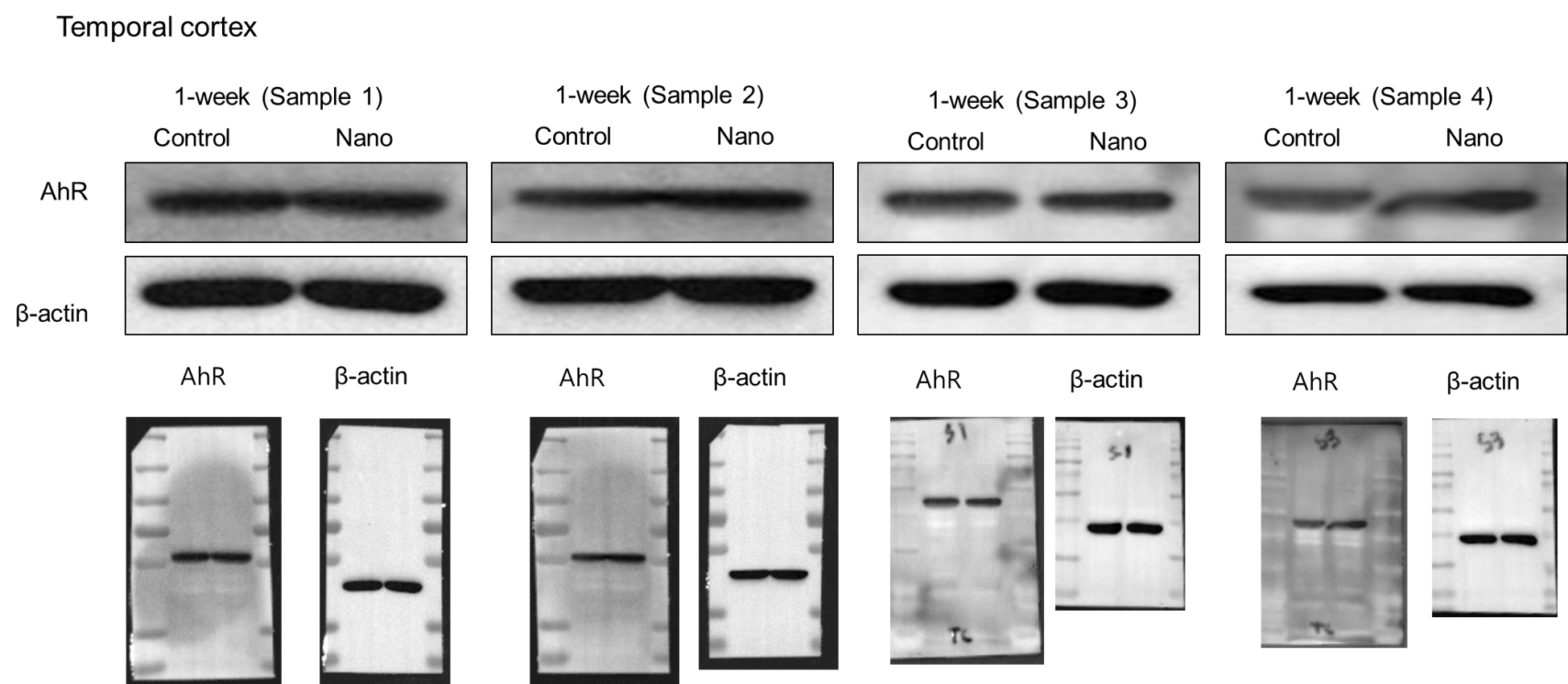

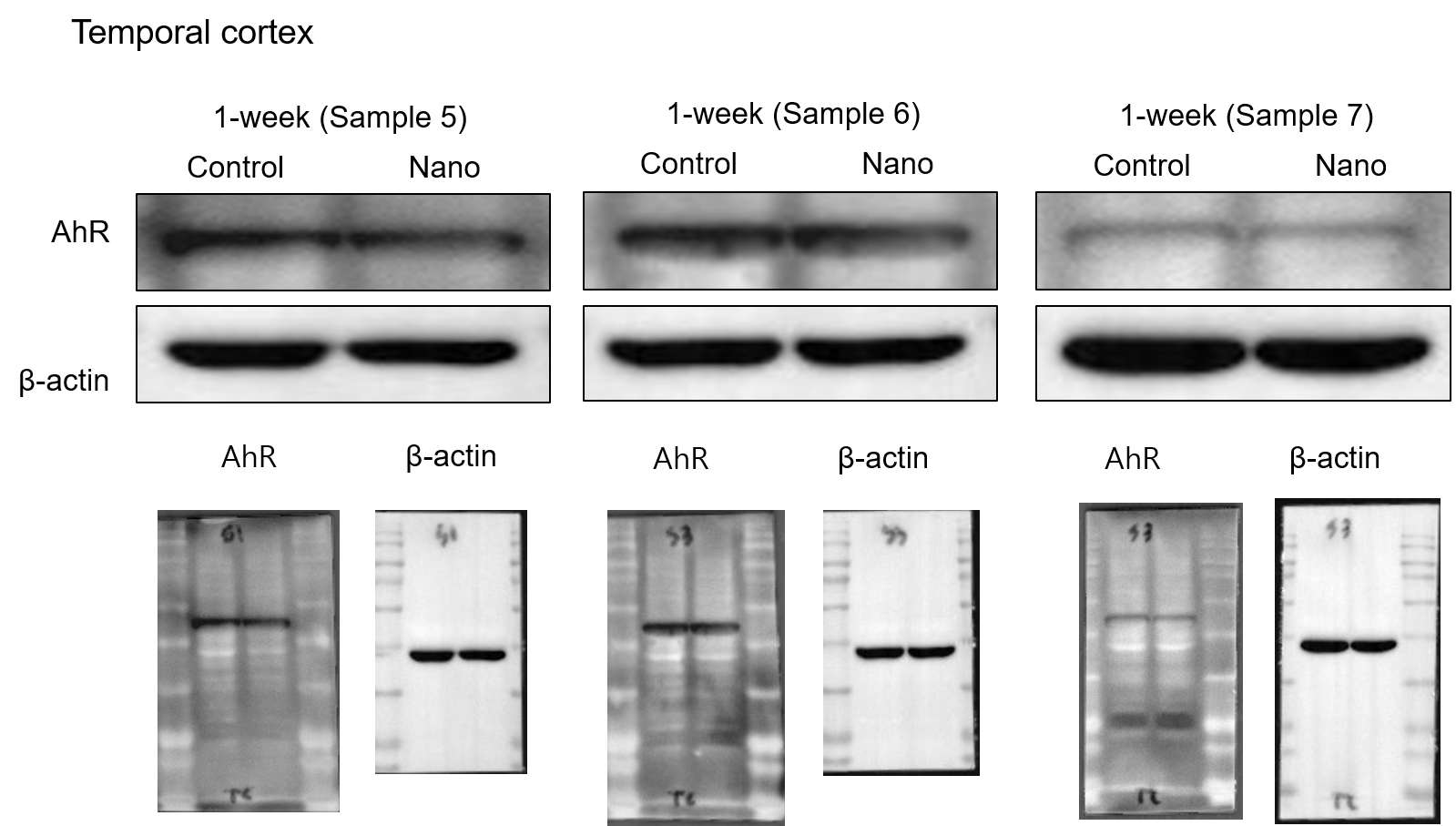

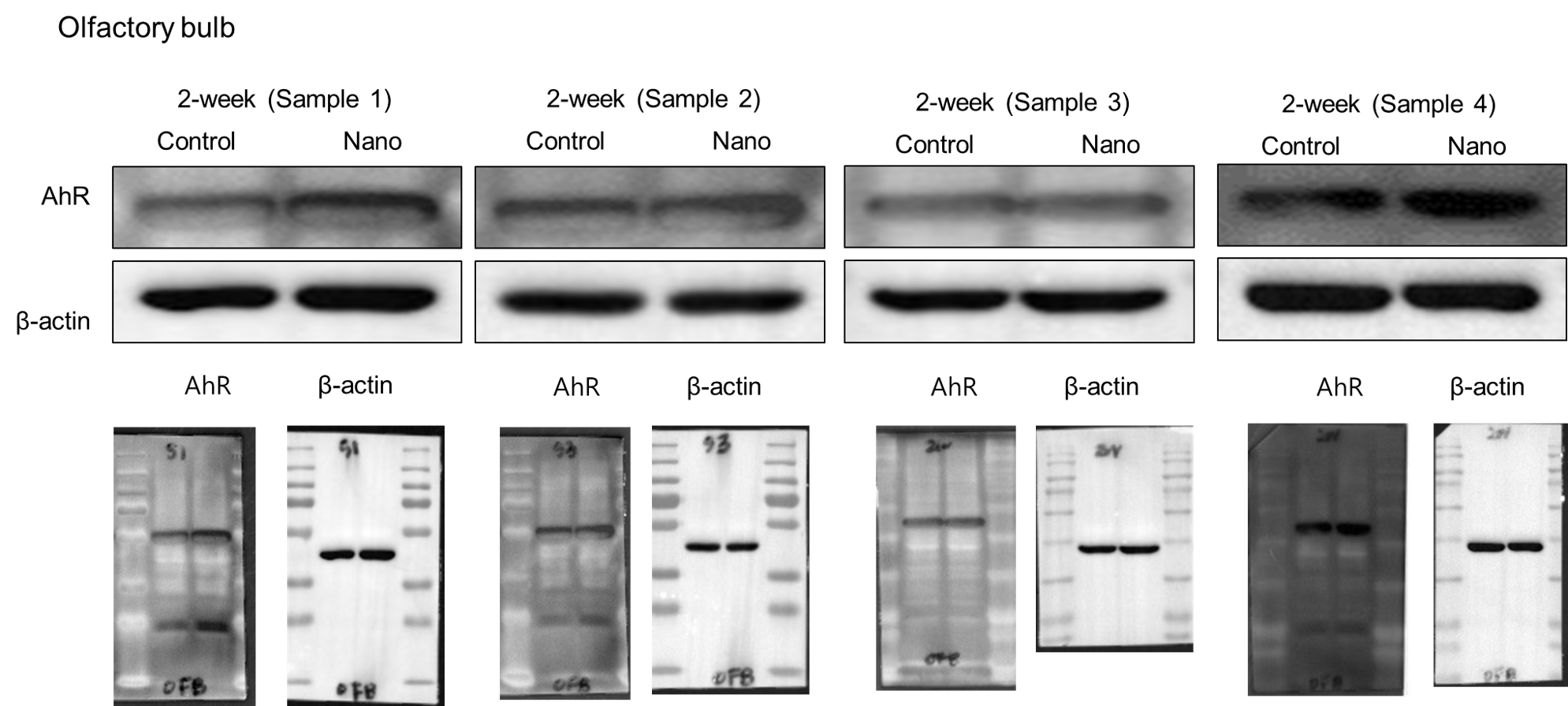

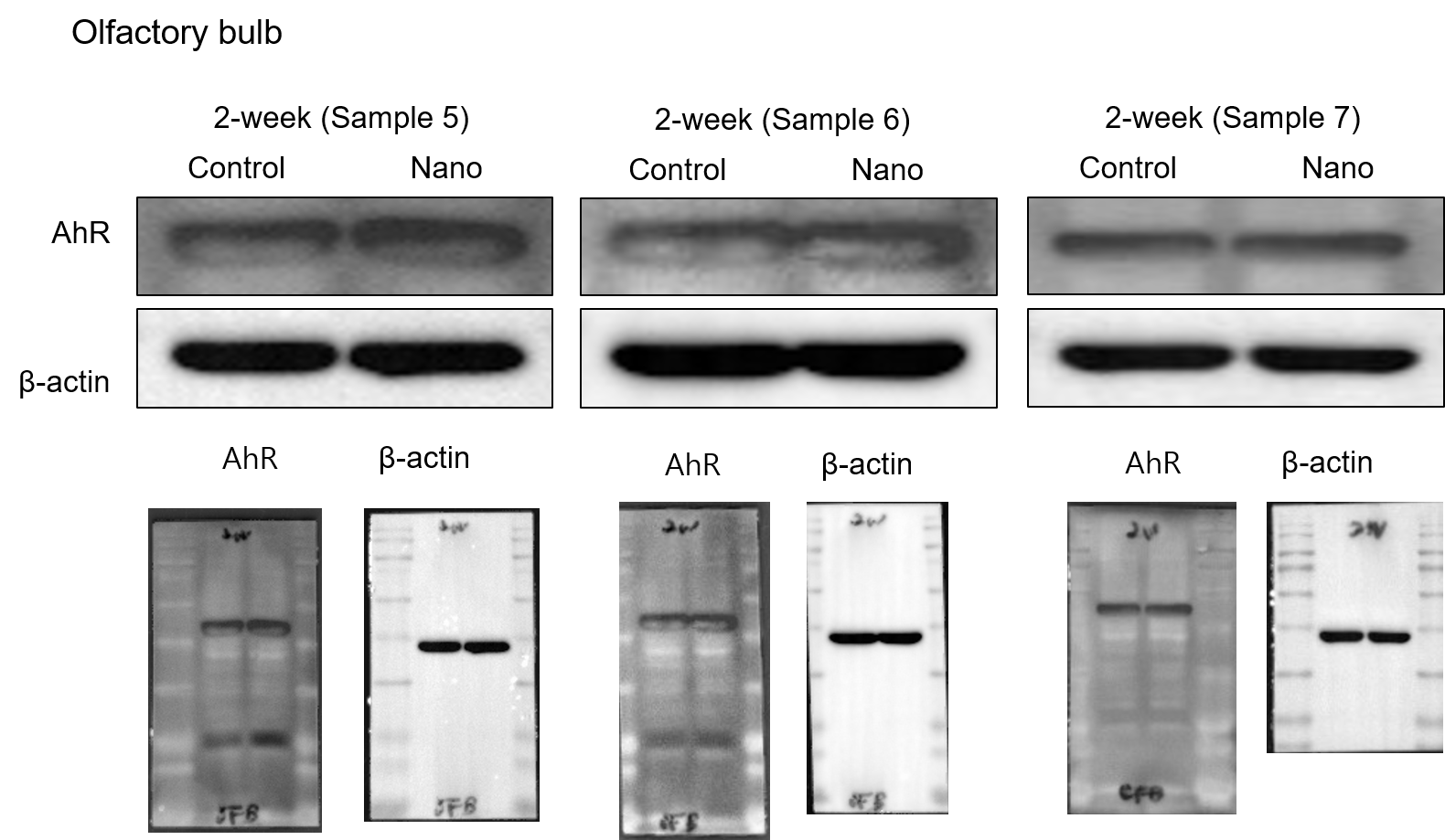

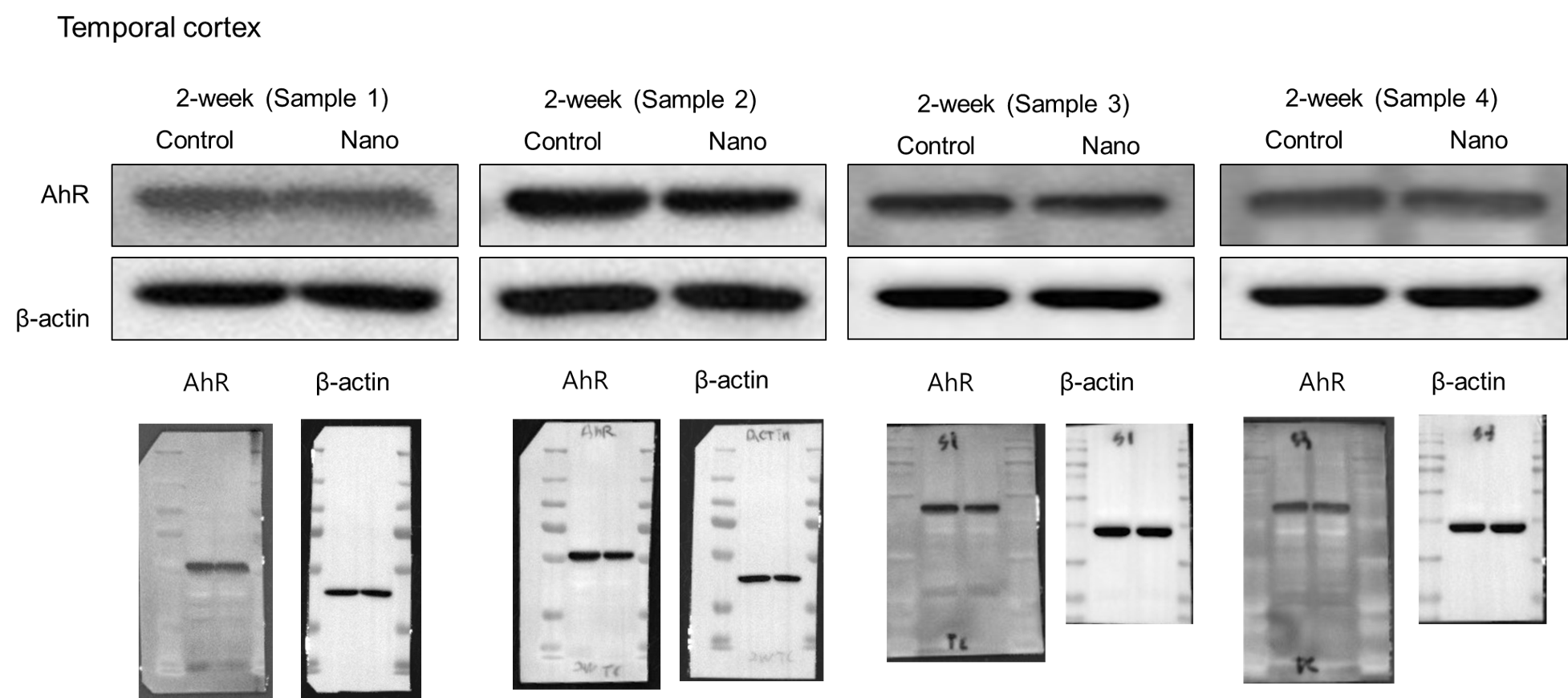

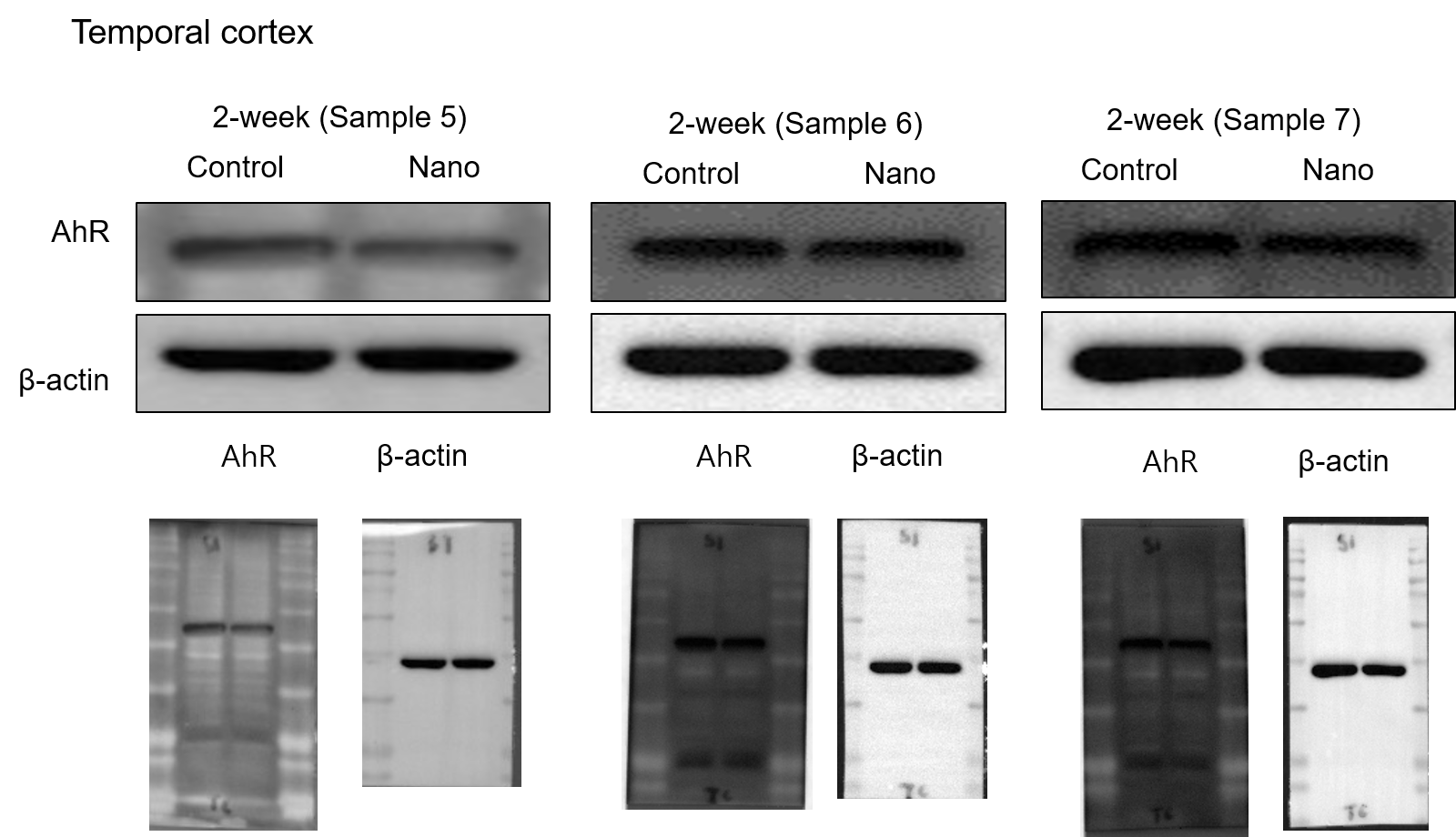

Supplement: Supplementary file 1 — Supplementary Information. [file 41598_2021_96593_MOESM1_ESM.docx]
